# Packing polymorphism in the crystal structure of 4,5-dimeth­oxy-2-nitro­benzyl acetate

**DOI:** 10.1107/S2056989015006714

**Published:** 2015-04-11

**Authors:** Noriko Chikaraishi Kasuga, Yusuke Saito, Hiroyasu Sato, Kazuo Yamaguchi

**Affiliations:** a Department of Materials Science, Faculty of Science, Kanagawa University, Tsuchiya, Hiratsuka, Kanagawa 259-1293, Japan; b Rigaku Corporation 3-9-12 Matsubara-cho, Akishima, Tokyo 196-8666, Japan

**Keywords:** crystal structure, packing polymorphism, 2-nitro­benzyl ester, π–π inter­actions, C—H⋯O inter­actions

## Abstract

The title compound, C_11_H_13_NO_6_, shows two polymorphs, orange and yellow forms, both of which crystallize in the space group *P*2_1_/*c*. The mol­ecular structures in the two polymorphs are essentially similar and adopt a planar structure, the maximum deviations for the non-H atoms being 0.1836 (13) and 0.1276 (13) Å, respectively, for the orange and yellow forms. In the orange crystal, mol­ecules are linked by an inter­molecular C—H⋯O inter­action into a helical chain along the *b-*axis direction. The chains are stacked along the *c* axis through a π–π inter­action [centroid–centroid distance = 3.6087 (11) Å], forming a layer parallel to the *bc* plane. In the yellow crystal, mol­ecules are connected through C—H⋯O inter­actions into a sheet structure parallel to (-302). No significant π–π inter­action is observed. The unit-cell volume of the orange crystal is larger than that of the yellow one, and this accounts for the predominant growth of the yellow crystal.

## Abstract 2

The title compound shows two packing polymorphs, in which the mol­ecular structures are planar and essentially similar. One crystal shows inter­molecular C—H⋯O and π–π inter­actions, while the other crystal exhibits several modes of inter­molecular C—H⋯O inter­actions.

## Chemical context   

Polymorphism is of inter­est in crystallization, phase transition, material synthesis and the pharmaceutical industry because differences in the crystal packing and/or conformation of compounds with the same formula can change the chemical and physical properties, including solubility, bioavailability and so forth (Moulton & Zaworotko, 2001 ▸; Matsuo & Matsuoka, 2007 ▸; Yu, 2010 ▸). We have been investigating silane coupling agents and thiols with distal functional groups protected by photolabile 2-nitro­benzyl groups (Edagawa *et al.*, 2012 ▸). During the course of photoremoval studies of these materials, we found that the simple ester, 4,5-dimeth­oxy-2-nitro­benzyl acetate, which releases acetic acid on photo-irradiation, forms two different types of crystals, orange rods and yellow needles. Here, we report the crystal structures of these two polymorphs of the title compound.
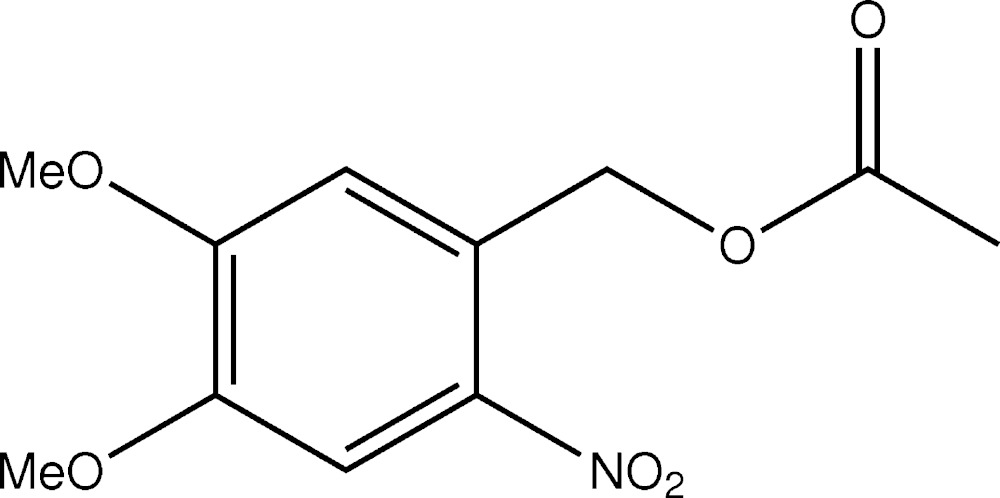


## Structural commentary   

**Figure 1:**
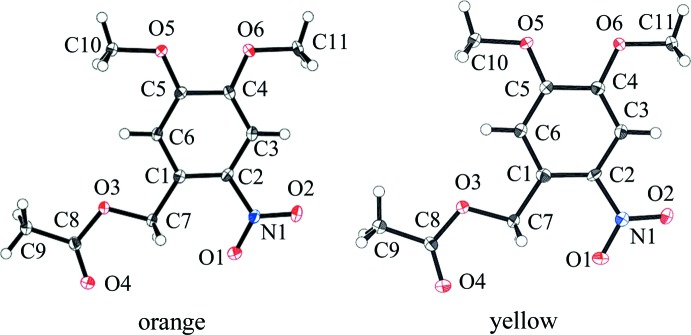
The mol­ecular structures of the title compound polymorphs, with atom labelling. Displacement ellipsoids are drawn at the 50% probability level.

The mol­ecular structures of the two crystals are approximately planar and almost identical, as shown in Fig. 1 ▸. The C2—C1—C7—O3, C9—C8—O3—C7, C5—C4—O5—C10 and C4—C5—O6—C11 torsion angles in the two crystals are approximately 180°. The dihedral angles between the benzene ring (C1–C6) and the nitro group (O1/N1/O2) are 9.54 (11) and 4.15 (7)° for the orange and yellow polymorphs, respectively.

## Supra­molecular features   

**Figure 2:**
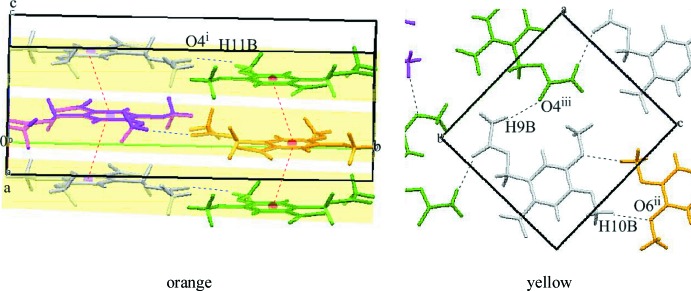
Inter­molecular C—H⋯O (black dashed lines) and π–π (red dashed lines) inter­actions in the orange crystal (left), and inter­molecular C—H⋯O inter­actions (black dashed lines) between meth­oxy groups and between acetyl groups in the yellow crystal (right). [Symmetry codes: (i) 1 − *x*, −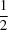 + *y*, 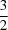 − *z*; (ii) 1 − *x*, 1 − *y*, 2 − *z*; (iii) −*x*, −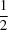 + *y*, 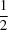 − *z*.]

Although the two crystals crystallize in the same space group (*P*2_1_/*c*) with *Z′* = 1, their packing modes are different. In the orange crystal, the mol­ecules are connected by an inter­molecular C—H⋯O inter­action [C11—H11*B*⋯O4^i^; symmetry code: (i) 1 − *x*, −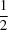 + *y*, 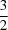 − *z*; Table 1 ▸] between the meth­oxy group and the carbonyl group, forming a helical chain along the *b* axis as shown in Fig. 2 ▸, left. In addition, a π–π inter­action between the benzene rings with a centroid–centroid distance of 3.6087 (11) Å links the chains to be stacked along the *c* axis. In the yellow crystal, the mol­ecules located in the plane perpendicular to the *ac* plane are connected by C—H⋯O inter­actions (Table 2 ▸) between meth­oxy groups [C10—H10*B*⋯O6^ii^; symmetry code: (ii) 1 − *x*, 1 − *y*, 2 − *z*] and between acetyl groups [C9—H9*B*⋯O4^iii^; symmetry code: (iii) −*x*, −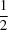 + *y*, 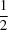 − *z*], forming a sheet structure parallel to (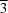
02) (Fig. 2 ▸, right).

**Figure 3:**
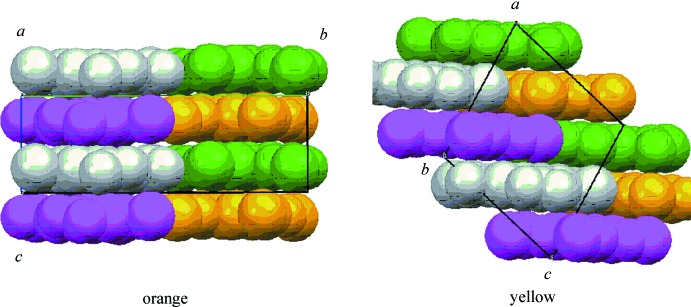
Side views of space-filling models of mol­ecular packing of the orange (left) and yellow (right) crystals.

In the orange crystal, the mol­ecules are stacked in columnar structures *via* π–π inter­actions along the *c* axis (Fig. 3 ▸, left). In contrast, no π–π inter­actions are observed in the yellow crystal. The mol­ecules are therefore terraced along the diagonal line of the *a* and *c* axes as shown in Fig. 3 ▸, right. As a result of these packing differences, the volume of the unit cell of the orange crystal is larger than that of the yellow one, *i.e.*, the orange crystal contains slightly more void space than the yellow one. This would account for the predominant growth of the yellow crystals.

## Synthesis and crystallization   

4,5-Dimeth­oxy-2-nitro­benzyl alcohol (0.714 g, 3.35 mmol), acetic anhydride (0.63 ml, 6.66 mmol), Et_3_N (1 ml) and CH_2_Cl_2_ (20 ml) were placed in a 100 mL flask, and the mixture was stirred at ambient temperature overnight. The mixture was extracted with CH_2_Cl_2_ (20 ml × 3), washed with brine, dried over MgSO_4_, and evaporated to give a yellow solid (0.773 g, 90% yield). The solid was crystallized by slow evaporation from a mixed solution of ethyl acetate and hexane (1:1). Orange crystals were occasionally obtained in small amounts, but the yellow crystals grew predominantly.

## Refinement   

Crystal data, data collection and structure refinement details are summarized in Table 3 ▸. All H atoms were located geometrically and refined using a riding model, with C—H = 0.99 Å and *U*_iso_(H) = 1.2*U*_eq_(C) for methyl­ene H atoms, C—H = 0.95 Å and *U*_iso_(H) = 1.2*U*_eq_(C) for aromatic H atoms, and C—H = 0.98 Å and *U*_iso_(H) = 1.5*U*_eq_(C) for methyl H atoms.

## Supplementary Material

Crystal structure: contains datablock(s) orange, yellow, global. DOI: 10.1107/S2056989015006714/is5391sup1.cif

Structure factors: contains datablock(s) orange. DOI: 10.1107/S2056989015006714/is5391orangesup2.hkl

Supporting information file. DOI: 10.1107/S2056989015006714/is5391orangesup4.cdx

Structure factors: contains datablock(s) yellow. DOI: 10.1107/S2056989015006714/is5391yellowsup3.hkl

Supporting information file. DOI: 10.1107/S2056989015006714/is5391yellowsup5.cdx

Supporting information file. DOI: 10.1107/S2056989015006714/is5391orangesup6.cml

Supporting information file. DOI: 10.1107/S2056989015006714/is5391yellowsup7.cml

CCDC references: 967703, 967704

Additional supporting information:  crystallographic information; 3D view; checkCIF report

## Figures and Tables

**Table 1:** Hydrogen-bond geometry (Å, °) for orange[Chem scheme1]

*D*—H⋯*A*	*D*—H	H⋯*A*	*D*⋯*A*	*D*—H⋯*A*
C11—H11*B*⋯O4^i^	0.98	2.50	3.369 (2)	147

Symmetry code: (i) 
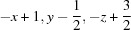
.

**Table 2:** Hydrogen-bond geometry (Å, °) for yellow[Chem scheme1]

*D*—H⋯*A*	*D*—H	H⋯*A*	*D*⋯*A*	*D*—H⋯*A*
C9—H9*B*⋯O4^iii^	0.98	2.40	3.375 (2)	174
C10—H10*B*⋯O6^ii^	0.98	2.51	3.472 (2)	169

Symmetry codes: (ii) 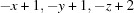; (iii) 
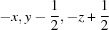
.

**Table 3:** Experimental details

	orange	yellow
Crystal data		
Chemical formula	C_11_H_13_NO_6_	C_11_H_13_NO_6_
*M*_r_	255.22	255.22
Crystal system, space group	Monoclinic, *P*2_1_/*c*	Monoclinic, *P*2_1_/*c*
Temperature (K)	93	93
*a*, *b*, *c* (Å)	8.8751 (13), 19.555 (2), 6.8688 (9)	10.476 (3), 10.714 (3), 10.266 (3)
β (°)	106.298 (6)	105.077 (10)
*V* (Å^3^)	1144.2 (3)	1112.6 (6)
*Z*	4	4
Radiation type	Mo *K*α	Mo *K*α
μ (mm^−1^)	0.12	0.13
Crystal size (mm)	0.45 × 0.42 × 0.39	0.56 × 0.54 × 0.25

Data collection		
Diffractometer	Rigaku Mercury375R	Rigaku Mercury375R
Absorption correction	Multi-scan (*REQAB*; Rigaku, 1998 ▸)	Multi-scan (*REQAB*; Rigaku, 1998 ▸)
*T*_min_, *T*_max_	0.960, 0.970	0.797, 0.970
No. of measured, independent and observed [*I* > 2σ(*I*)] reflections	11495, 2612, 2098	9498, 2058, 1769
*R*_int_	0.047	0.033
(sin θ/λ)_max_ (Å^−1^)	0.649	0.606

Refinement		
*R*[*F* ^2^ > 2σ(*F* ^2^)], *wR*(*F* ^2^), *S*	0.051, 0.132, 1.11	0.049, 0.130, 1.13
No. of reflections	2612	2058
No. of parameters	166	166
H-atom treatment	H-atom parameters not refined	H-atom parameters not refined
Δρ_max_, Δρ_min_ (e Å^−3^)	0.39, −0.30	0.38, −0.35

Computer programs: *CrystalClear-SM Expert* (Rigaku, 2011 ▸), *SIR2004* (Burla *et al.*, 2005 ▸), *SHELXL97* (Sheldrick, 2008 ▸), *Mercury* (Macrae *et al.*, 2008 ▸), *Yadokari-XG* (Wakita, 2001 ▸).

## supplementary crystallographic information

### (orange) 4,5-Dimethoxy-2-nitrobenzyl acetate Crystal data

**Table:**

C_11_H_13_NO_6_	*F*(000) = 536
*M**_r_* = 255.22	*D*_x_ = 1.48 Mg m^−^^3^
Monoclinic, *P*2_1_/*c*	Mo *K*α radiation, λ = 0.71069 Å
Hall symbol: -P 2ybc	Cell parameters from 2655 reflections
*a* = 8.8751 (13) Å	θ = 3.1–27.5°
*b* = 19.555 (2) Å	µ = 0.12 mm^−^^1^
*c* = 6.8688 (9) Å	*T* = 93 K
β = 106.298 (6)°	Platelet, orange
*V* = 1144.2 (3) Å^3^	0.45 × 0.42 × 0.39 mm
*Z* = 4	

### (orange) 4,5-Dimethoxy-2-nitrobenzyl acetate Data collection

**Table:**

Rigaku Mercury375R (2x2 bin mode) diffractometer	2612 independent reflections
Radiation source: fine-focus sealed tube	2098 reflections with *I* > 2σ(*I*)
Graphite monochromator	*R*_int_ = 0.047
Detector resolution: 13.6612 pixels mm^-1^	θ_max_ = 27.5°, θ_min_ = 3.2°
profile data from ω–scans	*h* = −11→11
Absorption correction: multi-scan (*REQAB*; Rigaku, 1998)	*k* = −25→25
*T*_min_ = 0.960, *T*_max_ = 0.970	*l* = −8→8
11495 measured reflections	

### (orange) 4,5-Dimethoxy-2-nitrobenzyl acetate Refinement

**Table:**

Refinement on *F*^2^	Primary atom site location: structure-invariant direct methods
Least-squares matrix: full	Secondary atom site location: difference Fourier map
*R*[*F*^2^ > 2σ(*F*^2^)] = 0.051	Hydrogen site location: inferred from neighbouring sites
*wR*(*F*^2^) = 0.132	H-atom parameters not refined
*S* = 1.11	*w* = 1/[σ^2^(*F*_o_^2^) + (0.0597*P*)^2^ + 0.5862*P*] where *P* = (*F*_o_^2^ + 2*F*_c_^2^)/3
2612 reflections	(Δ/σ)_max_ < 0.001
166 parameters	Δρ_max_ = 0.39 e Å^−^^3^
0 restraints	Δρ_min_ = −0.30 e Å^−^^3^

### (orange) 4,5-Dimethoxy-2-nitrobenzyl acetate Special details

**Table:**

Geometry. All e.s.d.'s (except the e.s.d. in the dihedral angle between two l.s. planes) are estimated using the full covariance matrix. The cell e.s.d.'s are taken into account individually in the estimation of e.s.d.'s in distances, angles and torsion angles; correlations between e.s.d.'s in cell parameters are only used when they are defined by crystal symmetry. An approximate (isotropic) treatment of cell e.s.d.'s is used for estimating e.s.d.'s involving l.s. planes.
Refinement. Refinement of *F*^2^ against ALL reflections. The weighted *R*-factor *wR* and goodness of fit *S* are based on *F*^2^, conventional *R*-factors *R* are based on *F*, with *F* set to zero for negative *F*^2^. The threshold expression of *F*^2^ > σ(*F*^2^) is used only for calculating *R*-factors(gt) *etc*. and is not relevant to the choice of reflections for refinement. *R*-factors based on *F*^2^ are statistically about twice as large as those based on *F*, and *R*- factors based on ALL data will be even larger.

### (orange) 4,5-Dimethoxy-2-nitrobenzyl acetate Fractional atomic coordinates and isotropic or equivalent isotropic displacement parameters (Å^2^)

**Table:**

	*x*	*y*	*z*	*U*_iso_*/*U*_eq_	
C1	0.19328 (19)	0.29434 (8)	0.7664 (2)	0.0124 (3)	
C2	0.04911 (19)	0.26435 (9)	0.7614 (3)	0.0139 (3)	
C3	0.02606 (19)	0.19342 (8)	0.7577 (3)	0.0140 (3)	
H3	−0.0739	0.1752	0.7541	0.017*	
C4	0.1487 (2)	0.15013 (8)	0.7593 (3)	0.0139 (3)	
C5	0.29755 (19)	0.17865 (8)	0.7678 (2)	0.0125 (3)	
C6	0.31701 (19)	0.24911 (8)	0.7700 (2)	0.0128 (3)	
H6	0.4171	0.2673	0.7740	0.015*	
C7	0.22141 (19)	0.37064 (8)	0.7674 (3)	0.0138 (3)	
H7A	0.1502	0.3919	0.6450	0.017*	
H7B	0.2010	0.3916	0.8887	0.017*	
C8	0.4225 (2)	0.44786 (9)	0.7575 (3)	0.0155 (4)	
C9	0.5882 (2)	0.45412 (9)	0.7490 (3)	0.0218 (4)	
H9A	0.5938	0.4425	0.6124	0.033*	
H9B	0.6550	0.4227	0.8475	0.033*	
H9C	0.6247	0.5012	0.7816	0.033*	
C10	−0.0084 (2)	0.05009 (9)	0.7387 (3)	0.0200 (4)	
H10A	−0.0436	0.0632	0.8565	0.030*	
H10B	0.0007	0.0002	0.7344	0.030*	
H10C	−0.0848	0.0660	0.6144	0.030*	
C11	0.5657 (2)	0.15867 (9)	0.7809 (3)	0.0187 (4)	
H11A	0.5598	0.1883	0.6639	0.028*	
H11B	0.6370	0.1205	0.7803	0.028*	
H11C	0.6051	0.1850	0.9063	0.028*	
N1	−0.08677 (17)	0.30611 (7)	0.7608 (2)	0.0154 (3)	
O1	−0.08009 (15)	0.36827 (7)	0.7379 (2)	0.0252 (3)	
O2	−0.20491 (15)	0.27751 (7)	0.7846 (2)	0.0236 (3)	
O3	0.38271 (14)	0.38139 (6)	0.7701 (2)	0.0154 (3)	
O4	0.33181 (16)	0.49408 (7)	0.7520 (2)	0.0257 (3)	
O5	0.14161 (14)	0.08062 (6)	0.7543 (2)	0.0176 (3)	
O6	0.41110 (14)	0.13243 (6)	0.7695 (2)	0.0164 (3)	

### (orange) 4,5-Dimethoxy-2-nitrobenzyl acetate Atomic displacement parameters (Å^2^)

**Table:**

	*U*^11^	*U*^22^	*U*^33^	*U*^12^	*U*^13^	*U*^23^
C1	0.0128 (8)	0.0155 (8)	0.0088 (8)	0.0001 (6)	0.0027 (6)	0.0011 (6)
C2	0.0112 (8)	0.0174 (8)	0.0137 (8)	0.0022 (6)	0.0046 (6)	0.0009 (6)
C3	0.0121 (8)	0.0174 (8)	0.0127 (8)	−0.0021 (6)	0.0037 (6)	0.0004 (6)
C4	0.0147 (8)	0.0137 (8)	0.0133 (8)	−0.0031 (6)	0.0040 (6)	0.0003 (6)
C5	0.0134 (8)	0.0152 (8)	0.0095 (8)	0.0014 (6)	0.0041 (6)	−0.0001 (6)
C6	0.0113 (8)	0.0160 (8)	0.0117 (8)	−0.0008 (6)	0.0043 (6)	−0.0002 (6)
C7	0.0105 (8)	0.0147 (8)	0.0177 (9)	−0.0003 (6)	0.0065 (6)	0.0002 (6)
C8	0.0161 (8)	0.0149 (8)	0.0164 (9)	−0.0025 (6)	0.0063 (7)	−0.0006 (6)
C9	0.0140 (8)	0.0182 (9)	0.0347 (11)	−0.0021 (7)	0.0094 (8)	−0.0009 (8)
C10	0.0173 (9)	0.0163 (8)	0.0271 (10)	−0.0074 (7)	0.0075 (7)	−0.0016 (7)
C11	0.0120 (8)	0.0170 (8)	0.0277 (10)	−0.0006 (6)	0.0065 (7)	−0.0011 (7)
N1	0.0113 (7)	0.0171 (7)	0.0177 (8)	−0.0005 (5)	0.0041 (6)	−0.0007 (5)
O1	0.0179 (7)	0.0159 (6)	0.0437 (9)	0.0031 (5)	0.0119 (6)	0.0037 (6)
O2	0.0133 (6)	0.0236 (7)	0.0364 (8)	−0.0016 (5)	0.0111 (6)	0.0010 (6)
O3	0.0114 (6)	0.0131 (6)	0.0227 (7)	−0.0011 (4)	0.0063 (5)	0.0000 (5)
O4	0.0203 (7)	0.0141 (6)	0.0461 (9)	0.0008 (5)	0.0147 (6)	0.0018 (6)
O5	0.0160 (6)	0.0120 (6)	0.0264 (7)	−0.0022 (5)	0.0085 (5)	−0.0001 (5)
O6	0.0124 (6)	0.0137 (6)	0.0244 (7)	0.0013 (5)	0.0071 (5)	0.0005 (5)

### (orange) 4,5-Dimethoxy-2-nitrobenzyl acetate Geometric parameters (Å, º)

**Table:**

C1—C2	1.399 (2)	C8—O3	1.356 (2)
C1—C6	1.405 (2)	C8—C9	1.494 (2)
C1—C7	1.512 (2)	C9—H9A	0.9800
C2—C3	1.401 (2)	C9—H9B	0.9800
C2—N1	1.456 (2)	C9—H9C	0.9800
C3—C4	1.377 (2)	C10—O5	1.436 (2)
C3—H3	0.9500	C10—H10A	0.9800
C4—O5	1.361 (2)	C10—H10B	0.9800
C4—C5	1.420 (2)	C10—H10C	0.9800
C5—O6	1.351 (2)	C11—O6	1.446 (2)
C5—C6	1.388 (2)	C11—H11A	0.9800
C6—H6	0.9500	C11—H11B	0.9800
C7—O3	1.4419 (19)	C11—H11C	0.9800
C7—H7A	0.9900	N1—O1	1.229 (2)
C7—H7B	0.9900	N1—O2	1.2390 (19)
C8—O4	1.204 (2)		
			
C2—C1—C6	116.20 (15)	O3—C8—C9	110.96 (15)
C2—C1—C7	124.21 (15)	C8—C9—H9A	109.5
C6—C1—C7	119.59 (15)	C8—C9—H9B	109.5
C1—C2—C3	122.90 (15)	H9A—C9—H9B	109.5
C1—C2—N1	121.08 (15)	C8—C9—H9C	109.5
C3—C2—N1	116.02 (15)	H9A—C9—H9C	109.5
C4—C3—C2	119.83 (15)	H9B—C9—H9C	109.5
C4—C3—H3	120.1	O5—C10—H10A	109.5
C2—C3—H3	120.1	O5—C10—H10B	109.5
O5—C4—C3	125.67 (15)	H10A—C10—H10B	109.5
O5—C4—C5	115.43 (15)	O5—C10—H10C	109.5
C3—C4—C5	118.90 (15)	H10A—C10—H10C	109.5
O6—C5—C6	125.00 (15)	H10B—C10—H10C	109.5
O6—C5—C4	114.87 (15)	O6—C11—H11A	109.5
C6—C5—C4	120.12 (15)	O6—C11—H11B	109.5
C5—C6—C1	122.03 (15)	H11A—C11—H11B	109.5
C5—C6—H6	119.0	O6—C11—H11C	109.5
C1—C6—H6	119.0	H11A—C11—H11C	109.5
O3—C7—C1	107.82 (13)	H11B—C11—H11C	109.5
O3—C7—H7A	110.1	O1—N1—O2	122.43 (15)
C1—C7—H7A	110.1	O1—N1—C2	119.04 (14)
O3—C7—H7B	110.1	O2—N1—C2	118.53 (14)
C1—C7—H7B	110.1	C8—O3—C7	114.47 (13)
H7A—C7—H7B	108.5	C4—O5—C10	116.93 (13)
O4—C8—O3	122.58 (16)	C5—O6—C11	117.20 (13)
O4—C8—C9	126.45 (16)		
			
C6—C1—C2—C3	−0.7 (2)	C7—C1—C6—C5	−179.55 (15)
C7—C1—C2—C3	179.08 (16)	C2—C1—C7—O3	−179.18 (15)
C6—C1—C2—N1	178.95 (15)	C6—C1—C7—O3	0.6 (2)
C7—C1—C2—N1	−1.2 (3)	C1—C2—N1—O1	9.5 (2)
C1—C2—C3—C4	0.1 (3)	C3—C2—N1—O1	−170.83 (16)
N1—C2—C3—C4	−179.59 (15)	C1—C2—N1—O2	−170.13 (16)
C2—C3—C4—O5	−179.39 (16)	C3—C2—N1—O2	9.6 (2)
C2—C3—C4—C5	1.0 (2)	O4—C8—O3—C7	2.5 (2)
O5—C4—C5—O6	0.1 (2)	C9—C8—O3—C7	−176.69 (15)
C3—C4—C5—O6	179.78 (15)	C1—C7—O3—C8	175.79 (14)
O5—C4—C5—C6	178.91 (14)	C3—C4—O5—C10	2.4 (3)
C3—C4—C5—C6	−1.4 (2)	C5—C4—O5—C10	−177.96 (15)
O6—C5—C6—C1	179.47 (15)	C6—C5—O6—C11	2.1 (2)
C4—C5—C6—C1	0.8 (2)	C4—C5—O6—C11	−179.17 (15)
C2—C1—C6—C5	0.3 (2)		

### (orange) 4,5-Dimethoxy-2-nitrobenzyl acetate Hydrogen-bond geometry (Å, º)

**Table:**

*D*—H···*A*	*D*—H	H···*A*	*D*···*A*	*D*—H···*A*
C11—H11*B*···O4^i^	0.98	2.50	3.369 (2)	147

Symmetry code: (i) −*x*+1, *y*−1/2, −*z*+3/2.

### (yellow) 4,5-Dimethoxy-2-nitrobenzyl Acetate Crystal data

**Table:**

C_11_H_13_NO_6_	*F*(000) = 536
*M**_r_* = 255.22	*D*_x_ = 1.52 Mg m^−^^3^
Monoclinic, *P*2_1_/*c*	Mo *K*α radiation, λ = 0.71069 Å
Hall symbol: -P 2ybc	Cell parameters from 2424 reflections
*a* = 10.476 (3) Å	θ = 3.1–27.5°
*b* = 10.714 (3) Å	µ = 0.13 mm^−^^1^
*c* = 10.266 (3) Å	*T* = 93 K
β = 105.077 (10)°	Neecle, yellow
*V* = 1112.6 (6) Å^3^	0.56 × 0.54 × 0.25 mm
*Z* = 4	

### (yellow) 4,5-Dimethoxy-2-nitrobenzyl Acetate Data collection

**Table:**

Rigaku Mercury375R (2x2 bin mode) diffractometer	2058 independent reflections
Radiation source: fine-focus sealed tube	1769 reflections with *I* > 2σ(*I*)
Graphite monochromator	*R*_int_ = 0.033
Detector resolution: 13.6612 pixels mm^-1^	θ_max_ = 25.5°, θ_min_ = 3.1°
profile data from ω–scan	*h* = −12→12
Absorption correction: multi-scan (*REQAB*; Rigaku, 1998)	*k* = −12→12
*T*_min_ = 0.797, *T*_max_ = 0.970	*l* = −12→12
9498 measured reflections	

### (yellow) 4,5-Dimethoxy-2-nitrobenzyl Acetate Refinement

**Table:**

Refinement on *F*^2^	Primary atom site location: structure-invariant direct methods
Least-squares matrix: full	Secondary atom site location: difference Fourier map
*R*[*F*^2^ > 2σ(*F*^2^)] = 0.049	Hydrogen site location: inferred from neighbouring sites
*wR*(*F*^2^) = 0.130	H-atom parameters not refined
*S* = 1.13	*w* = 1/[σ^2^(*F*_o_^2^) + (0.0689*P*)^2^ + 0.4454*P*] where *P* = (*F*_o_^2^ + 2*F*_c_^2^)/3
2058 reflections	(Δ/σ)_max_ < 0.001
166 parameters	Δρ_max_ = 0.38 e Å^−^^3^
0 restraints	Δρ_min_ = −0.35 e Å^−^^3^

### (yellow) 4,5-Dimethoxy-2-nitrobenzyl Acetate Special details

**Table:**

Experimental. Rigaku (1998). REQAB. Rigaku Corporation, Tokyo, Japan.
Geometry. All e.s.d.'s (except the e.s.d. in the dihedral angle between two l.s. planes) are estimated using the full covariance matrix. The cell e.s.d.'s are taken into account individually in the estimation of e.s.d.'s in distances, angles and torsion angles; correlations between e.s.d.'s in cell parameters are only used when they are defined by crystal symmetry. An approximate (isotropic) treatment of cell e.s.d.'s is used for estimating e.s.d.'s involving l.s. planes.
Refinement. Refinement of *F*^2^ against ALL reflections. The weighted *R*-factor *wR* and goodness of fit *S* are based on *F*^2^, conventional *R*-factors *R* are based on *F*, with *F* set to zero for negative *F*^2^. The threshold expression of *F*^2^ > σ(*F*^2^) is used only for calculating *R*-factors(gt) *etc*. and is not relevant to the choice of reflections for refinement. *R*-factors based on *F*^2^ are statistically about twice as large as those based on *F*, and *R*- factors based on ALL data will be even larger.

### (yellow) 4,5-Dimethoxy-2-nitrobenzyl Acetate Fractional atomic coordinates and isotropic or equivalent isotropic displacement parameters (Å^2^)

**Table:**

	*x*	*y*	*z*	*U*_iso_*/*U*_eq_	
C1	0.22297 (15)	0.82305 (16)	0.58154 (16)	0.0158 (4)	
C2	0.29963 (16)	0.88543 (15)	0.69423 (17)	0.0154 (4)	
C3	0.37895 (16)	0.82230 (16)	0.80564 (16)	0.0168 (4)	
H3	0.4307	0.8681	0.8801	0.020*	
C4	0.38181 (16)	0.69396 (16)	0.80717 (16)	0.0167 (4)	
C5	0.30317 (15)	0.62775 (16)	0.69555 (17)	0.0154 (4)	
C6	0.22676 (16)	0.69258 (16)	0.58567 (16)	0.0158 (4)	
H6	0.1754	0.6469	0.5109	0.019*	
C7	0.13844 (16)	0.88840 (15)	0.45857 (17)	0.0166 (4)	
H7A	0.0754	0.9456	0.4852	0.020*	
H7B	0.1948	0.9379	0.4140	0.020*	
C8	−0.01205 (16)	0.83679 (16)	0.25084 (16)	0.0175 (4)	
C9	−0.08377 (18)	0.73342 (16)	0.16403 (18)	0.0216 (4)	
H9A	−0.1781	0.7378	0.1603	0.032*	
H9B	−0.0481	0.6529	0.2022	0.032*	
H9C	−0.0721	0.7418	0.0728	0.032*	
C10	0.53711 (16)	0.68572 (16)	1.02246 (16)	0.0186 (4)	
H10A	0.6026	0.7354	0.9923	0.028*	
H10B	0.5826	0.6245	1.0895	0.028*	
H10C	0.4834	0.7409	1.0630	0.028*	
C11	0.22990 (18)	0.43083 (16)	0.59702 (17)	0.0211 (4)	
H11A	0.1368	0.4538	0.5832	0.032*	
H11B	0.2410	0.3417	0.6185	0.032*	
H11C	0.2574	0.4480	0.5146	0.032*	
O1	0.23683 (12)	1.08224 (11)	0.60592 (12)	0.0219 (3)	
O2	0.36433 (12)	1.07109 (11)	0.80903 (12)	0.0228 (3)	
O3	0.06713 (11)	0.79354 (11)	0.36698 (12)	0.0186 (3)	
O4	−0.02320 (12)	0.94638 (11)	0.22241 (12)	0.0229 (3)	
O5	0.45330 (11)	0.62196 (11)	0.90924 (12)	0.0183 (3)	
O6	0.30975 (12)	0.50251 (11)	0.70663 (12)	0.0187 (3)	
N1	0.30069 (14)	1.02142 (14)	0.70366 (14)	0.0175 (3)	

### (yellow) 4,5-Dimethoxy-2-nitrobenzyl Acetate Atomic displacement parameters (Å^2^)

**Table:**

	*U*^11^	*U*^22^	*U*^33^	*U*^12^	*U*^13^	*U*^23^
C1	0.0163 (8)	0.0161 (8)	0.0155 (8)	0.0025 (6)	0.0052 (7)	0.0004 (6)
C2	0.0196 (8)	0.0084 (8)	0.0189 (9)	0.0000 (6)	0.0064 (7)	−0.0011 (6)
C3	0.0179 (8)	0.0159 (8)	0.0154 (8)	−0.0026 (7)	0.0023 (7)	−0.0015 (6)
C4	0.0189 (8)	0.0155 (9)	0.0149 (8)	0.0008 (7)	0.0031 (7)	0.0008 (6)
C5	0.0163 (8)	0.0136 (9)	0.0158 (8)	−0.0006 (6)	0.0033 (7)	−0.0002 (6)
C6	0.0177 (8)	0.0137 (9)	0.0153 (8)	−0.0009 (6)	0.0033 (7)	−0.0023 (6)
C7	0.0193 (8)	0.0115 (8)	0.0163 (8)	−0.0006 (6)	0.0000 (7)	−0.0022 (6)
C8	0.0169 (8)	0.0188 (9)	0.0148 (8)	0.0001 (7)	0.0006 (7)	0.0014 (7)
C9	0.0233 (9)	0.0158 (9)	0.0212 (9)	0.0007 (7)	−0.0025 (7)	0.0003 (7)
C10	0.0199 (8)	0.0181 (9)	0.0144 (8)	−0.0017 (7)	−0.0018 (7)	−0.0009 (7)
C11	0.0278 (9)	0.0134 (9)	0.0188 (9)	−0.0016 (7)	0.0000 (7)	−0.0029 (6)
O1	0.0277 (7)	0.0145 (6)	0.0204 (7)	0.0032 (5)	0.0009 (5)	0.0035 (5)
O2	0.0307 (7)	0.0153 (7)	0.0189 (7)	−0.0016 (5)	−0.0001 (5)	−0.0052 (5)
O3	0.0215 (6)	0.0127 (6)	0.0173 (6)	0.0004 (5)	−0.0027 (5)	−0.0002 (5)
O4	0.0277 (7)	0.0140 (6)	0.0229 (7)	0.0001 (5)	−0.0008 (5)	0.0031 (5)
O5	0.0222 (6)	0.0131 (6)	0.0146 (6)	−0.0007 (5)	−0.0040 (5)	0.0008 (5)
O6	0.0245 (6)	0.0095 (6)	0.0183 (6)	−0.0001 (5)	−0.0013 (5)	−0.0001 (4)
N1	0.0195 (7)	0.0153 (8)	0.0168 (7)	−0.0007 (6)	0.0029 (6)	−0.0007 (6)

### (yellow) 4,5-Dimethoxy-2-nitrobenzyl Acetate Geometric parameters (Å, º)

**Table:**

C1—C2	1.395 (2)	C8—O3	1.345 (2)
C1—C6	1.399 (2)	C8—C9	1.496 (2)
C1—C7	1.512 (2)	C9—H9A	0.9800
C2—C3	1.401 (2)	C9—H9B	0.9800
C2—N1	1.460 (2)	C9—H9C	0.9800
C3—C4	1.375 (3)	C10—O5	1.4344 (19)
C3—H3	0.9500	C10—H10A	0.9800
C4—O5	1.359 (2)	C10—H10B	0.9800
C4—C5	1.416 (2)	C10—H10C	0.9800
C5—O6	1.347 (2)	C11—O6	1.437 (2)
C5—C6	1.388 (2)	C11—H11A	0.9800
C6—H6	0.9500	C11—H11B	0.9800
C7—O3	1.4524 (19)	C11—H11C	0.9800
C7—H7A	0.9900	O1—N1	1.2368 (19)
C7—H7B	0.9900	O2—N1	1.2339 (19)
C8—O4	1.208 (2)		
			
C2—C1—C6	116.61 (15)	O3—C8—C9	111.81 (14)
C2—C1—C7	123.79 (16)	C8—C9—H9A	109.5
C6—C1—C7	119.60 (14)	C8—C9—H9B	109.5
C1—C2—C3	122.48 (16)	H9A—C9—H9B	109.5
C1—C2—N1	121.72 (15)	C8—C9—H9C	109.5
C3—C2—N1	115.79 (15)	H9A—C9—H9C	109.5
C4—C3—C2	119.91 (15)	H9B—C9—H9C	109.5
C4—C3—H3	120.0	O5—C10—H10A	109.5
C2—C3—H3	120.0	O5—C10—H10B	109.5
O5—C4—C3	125.62 (15)	H10A—C10—H10B	109.5
O5—C4—C5	115.33 (15)	O5—C10—H10C	109.5
C3—C4—C5	119.04 (15)	H10A—C10—H10C	109.5
O6—C5—C6	124.98 (15)	H10B—C10—H10C	109.5
O6—C5—C4	115.13 (14)	O6—C11—H11A	109.5
C6—C5—C4	119.89 (16)	O6—C11—H11B	109.5
C5—C6—C1	122.05 (15)	H11A—C11—H11B	109.5
C5—C6—H6	119.0	O6—C11—H11C	109.5
C1—C6—H6	119.0	H11A—C11—H11C	109.5
O3—C7—C1	107.90 (13)	H11B—C11—H11C	109.5
O3—C7—H7A	110.1	C8—O3—C7	115.31 (13)
C1—C7—H7A	110.1	C4—O5—C10	116.94 (13)
O3—C7—H7B	110.1	C5—O6—C11	117.37 (13)
C1—C7—H7B	110.1	O2—N1—O1	122.61 (15)
H7A—C7—H7B	108.4	O2—N1—C2	118.78 (14)
O4—C8—O3	123.19 (15)	O1—N1—C2	118.60 (13)
O4—C8—C9	125.01 (15)		
			
C6—C1—C2—C3	1.2 (2)	C7—C1—C6—C5	179.61 (14)
C7—C1—C2—C3	−178.75 (15)	C2—C1—C7—O3	−176.32 (14)
C6—C1—C2—N1	−178.07 (14)	C6—C1—C7—O3	3.7 (2)
C7—C1—C2—N1	2.0 (2)	O4—C8—O3—C7	0.8 (2)
C1—C2—C3—C4	−0.8 (2)	C9—C8—O3—C7	−178.75 (13)
N1—C2—C3—C4	178.48 (15)	C1—C7—O3—C8	−179.24 (13)
C2—C3—C4—O5	−179.33 (14)	C3—C4—O5—C10	−2.8 (2)
C2—C3—C4—C5	−0.4 (2)	C5—C4—O5—C10	178.28 (13)
O5—C4—C5—O6	0.4 (2)	C6—C5—O6—C11	−1.0 (2)
C3—C4—C5—O6	−178.65 (15)	C4—C5—O6—C11	178.91 (13)
O5—C4—C5—C6	−179.73 (14)	C1—C2—N1—O2	175.79 (14)
C3—C4—C5—C6	1.3 (2)	C3—C2—N1—O2	−3.5 (2)
O6—C5—C6—C1	179.03 (15)	C1—C2—N1—O1	−3.7 (2)
C4—C5—C6—C1	−0.9 (2)	C3—C2—N1—O1	177.02 (14)
C2—C1—C6—C5	−0.3 (2)		

### (yellow) 4,5-Dimethoxy-2-nitrobenzyl Acetate Hydrogen-bond geometry (Å, º)

**Table:**

*D*—H···*A*	*D*—H	H···*A*	*D*···*A*	*D*—H···*A*
C9—H9*B*···O4^i^	0.98	2.40	3.375 (2)	174
C10—H10*B*···O6^ii^	0.98	2.51	3.472 (2)	169

Symmetry codes: (i) −*x*, *y*−1/2, −*z*+1/2; (ii) −*x*+1, −*y*+1, −*z*+2.
